# A Machine Learning Approach to Differentiate Cold and Hot Syndrome in Viral Pneumonia Integrating Traditional Chinese Medicine and Modern Medicine: Machine Learning Model Development and Validation

**DOI:** 10.2196/64725

**Published:** 2025-07-16

**Authors:** Xiaojie Jin, Yanru Wang, Jiarui Wang, Qian Gao, Yuhan Huang, Lingyu Shao, Jiali Zhao, Jintian Li, Ling Li, Zhiming Zhang, Shuyan Li, Yongqi Liu

**Affiliations:** 1 Key Laboratory of Dunhuang Medicine, Ministry of Education, Gansu University of Chinese Medicine, Dingxi East Road, 35th, Lanzhou, 730000, China, 86 13919019578; 2 College of Pharmacy, Gansu University of Chinese Medicine, Lanzhou, China; 3 The Second Affiliated Hospital of Xuzhou Medical University, Xuzhou, China; 4 School of Medical Information and Engineering, Xuzhou Medical University, Xuzhou, China; 5 Gansu Provincial Hospital of Traditional Chinese Medicine, Lanzhou, China

**Keywords:** syndrome differentiation, traditional Chinese medicine, viral pneumonia, dialectical treatment, machine learning, Chinese medicine, pneumonia, lungs, diagnostic model, retrospective study, artificial intelligence, China, model training, performance evaluation, laboratory tests, modern medicine

## Abstract

**Background:**

Syndrome differentiation in traditional Chinese medicine (TCM) is an ancient principle that guides disease diagnosis and treatment. Among these, the cold and hot syndromes play a crucial role in identifying the nature of the disease and guiding the treatment of viral pneumonia. However, differentiating between cold and hot syndromes is often considered esoteric. Machine learning offers a promising avenue for clinicians to identify these syndromes more accurately, thereby supporting more informed clinical decision-making in the treatment.

**Objective:**

This study aims to construct a diagnostic model for differentiating cold and hot syndromes in viral pneumonia by integrating TCM and modern medical features using machine learning methods.

**Methods:**

The application of 8 machine learning algorithms (gradient boosting machine [GBM], logistic regression, random forest, extreme gradient boosting [XGB], light gradient boosting machine [LGB], ridge regression, least absolute shrinkage and selection operator, and support vector machine) generated and validated (both internally and externally) a model for differentiating cold and hot syndromes in viral pneumonia, based on clinical data from 1484 patient samples collected at 2 medical centers between 2021 and 2022.

**Results:**

The GBM model, which combines TCM and modern medicine features, outperformed models using only TCM features or only modern medicine features in distinguishing cold and hot syndromes in patients with viral pneumonia. The optimal discrimination model comprised 13 optimal features (temperature, red cell distribution width-SD, creatinine, total bilirubin, globulin, C-reactive protein, unconjugated bilirubin, white blood cell, neutrophil percentage, aspartate transaminase/alanine transaminase, total cholesterol, thrombocytocrit, and age) and the GBM algorithm, achieving an area under the curve (AUC) of 0.7788. Under internal and external testing, the AUCs were 0.7645 and 0.8428, respectively. Moreover, significant differences were observed between the cold and hot syndrome groups in temperature (*P*=.02), red cell distribution width-SD (*P*<.001), neutrophil percentage (*P*=.01), total cholesterol (*P*=.003), thrombocytocrit (*P*<.001), and age (*P*<.001).

**Conclusions:**

This pioneering study integrates the theory of TCM cold and hot syndromes with modern laboratory-based tests through machine learning. The developed model offers a novel approach for differentiating cold and hot syndromes in viral pneumonia, enabling practitioners to identify the syndrome quickly and efficiently, thereby supporting more informed clinical decision-making. Additionally, this research provides new insights into the modernization and scientific interpretation of TCM syndrome differentiation.

## Introduction

Traditional Chinese medicine (TCM), a personalized and holistic approach, treats diseases using natural medical products tailored to a patient’s TCM syndrome patterns [1], that is, dialectical treatment of TCM. Specifically, TCM syndrome, a concept unique to TCM, is an abstraction of a variety of signs and symptoms. Dialectical treatment is the fundamental principle by which physicians diagnose, understand, and treat diseases. It involves comprehensively collecting patients’ clinical signs and symptoms through “inspection, auscultation, olfaction, inquiry, and palpation” to determine the TCM syndrome pattern. Subsequently, corresponding therapies, such as acupuncture, cupping, qigong, massage, and diet, are selected [2] to correct maladjustments and restore the body’s self-regulatory ability [3,4]. Guided by this unique principle, TCM has demonstrated remarkable clinical efficacy in treating various acute and chronic diseases, thus attracting increasing attention [5]. Especially in the fight against viral pneumonia, the importance of TCM has been widely recognized [6,7]. Consequently, promptly and accurately understanding the TCM syndrome pattern of a disease can reshape global health and welfare.

In recent years, with the successive outbreaks of SARS, influenza A (H1N1), influenza A (H7N9), COVID-19, and other viruses, viral pneumonia has become a sustained and widespread disease burden in many countries and globally. As with other diseases, the accurate identification of syndrome patterns is a prerequisite for the TCM-based prevention and treatment of viral pneumonia. Numerous studies have reported that the cold and hot syndromes are the pivotal TCM syndrome patterns in cases of viral pneumonia, such as SARS and COVID-19 [8-10]. Cold and hot syndromes, originating from the *Huangdi Neijing,* are 2 key components of the Eight Principle Syndromes—the general principles of dialectical treatment, which include cold and hot, sthenia and asthenia, exterior and interior, and yin and yang—and are used to identify the nature of a disease, reflecting disruptions in body homeostasis [11-13]. Cold syndrome–related expressions include coldness, cold pain, tastelessness, and clear, abundant urine, whereas hot syndrome is mainly characterized by heat, diaphoresis, a flushed face, burning pain, and deep-colored urine. The cold and hot syndrome framework has been widely applied in the diagnosis and treatment of viral pneumonia. Treating cold and hot syndromes within TCM has been shown to significantly alleviate clinical symptoms in patients with viral pneumonia [8,14]. For example, symptomatic treatments with Chinese medicines such as Lianhua Qingwen capsules and Xuanfeibaidu granules can markedly reduce the incidence of severe or critical events and improve clinical recovery [6,7], indicating that the prediction and differentiation of cold and hot syndromes play a vital role in the treatment of viral pneumonia.

Nevertheless, although clinical data and studies have indicated that TCM treatments for the cold and hot syndromes of viral pneumonia are effective, a major challenge remains in distinguishing between these syndromes—similar to the challenges in identifying other TCM syndrome patterns. Specifically, (1) the differentiation of cold and hot syndrome is recondite and lacks a solid scientific foundation in classical TCM theory, and (2) the accuracy of cold and hot syndrome diagnosis in TCM relies heavily on the physicians’ skills and years of clinical experience. Subjective factors play a decisive role in TCM diagnosis, which inevitably leads to a lack of standardized criteria and an objective diagnostic basis for cold and hot syndromes. This unique diagnostic model not only supports the open and divergent development of TCM, but also hinders the understanding of cold and hot syndromes in viral pneumonia from a modern biomedical perspective. It further restricts the domestic and international dissemination of TCM syndrome differentiation, making it one of the key bottlenecks in the development of TCM. Therefore, in the process of integrating TCM with modern medicine, it is worth considering how to combine TCM syndrome theory with modern medicine to enhance the scientific rigor and objectivity of cold and hot syndrome differentiation in the treatment of viral pneumonia.

Recently, with the rapid development of machine learning [15-18], artificial intelligence has brought unprecedented challenges and opportunities to the diagnosis of TCM syndrome. Interdisciplinary models combining TCM and artificial intelligence have been proposed to model TCM knowledge, diagnosis, and treatment in clinical practice. Among these, numerous models have been developed to simulate the syndrome differentiation process of human TCM doctors for automatic syndrome diagnosis. For example, Huang et al [19] successfully identified the liver-gallbladder dampness-heat syndrome in patients with breast cancer using a dataset based on the prescriptions of TCM clinical practitioners. Zhuo et al [20] effectively differentiated Qi deficiency blood stasis syndrome and phlegm stasis in channels syndrome in patients with stroke through artificial intelligence–assisted retinal feature analysis. These examples demonstrate that machine learning enables a modern interpretation of the scientific connotation of TCM syndrome differentiation, supporting improved clinical decision-making in disease treatment. However, currently, there is still a lack of machine learning–based differentiation of cold and hot syndromes in viral pneumonia.

Among the machine learning algorithms in artificial intelligence, gradient boosting machine (GBM), logistic regression (LR), random forest (RF), extreme gradient boosting (XGB), and light gradient boosting machine (LGB), among others, have demonstrated promising performance [21-24]. Consequently, this study aimed to compare 8 machine learning methods, namely, GBM, LR, RF, XGB, LGB, ridge regression (RIDGE), least absolute shrinkage and selection operator (LASSO), and support vector machine (SVM), by integrating laboratory test indicators from modern medicine with the TCM cold and hot syndrome, to explore the connection between them and construct a diagnostic model for differentiating the cold and hot syndromes in viral pneumonia. This approach seeks to provide a new diagnostic method for the clinical differentiation of cold and hot syndromes in viral pneumonia and to offer new perspectives for the scientization, standardization, and objectivity of TCM syndrome differentiation theory in guiding treatment.

## Methods

### Source of Materials

In this study, a retrospective review of case sheets was conducted on samples from 1401 patients diagnosed with viral pneumonia between January 18 and November 6, 2021, at the Second People’s Hospital of Lanzhou City in China. Samples were excluded based on the following criteria: (1) the diagnosis was not made using the Eight Principle Syndromes of TCM, and (2) the patient was diagnosed with both cold and hot syndrome patterns simultaneously. A total of 602 samples met the inclusion criteria. Samples with more than 20% (ie, 19/93 features) missing values were further excluded, resulting in 382 samples ultimately included for analysis. These comprised 97 negative samples (cold syndrome) and 285 positive samples (hot syndrome). Additionally, a stratified sampling strategy was applied to split the original dataset into training and internal test cohorts at an 8:2 ratio (details are provided in the “Performance Evaluation and Model Validation” section).

The external test cohort consisted of 83 patients with viral pneumonia diagnosed with either cold or hot syndrome at Lanzhou Heavy Ion Hospital between July and August 2022. This cohort included 36 patients with the hot syndrome and 47 patients with the cold syndrome.

### TCM and Modern Medicine Features Collection and Cold and Hot Syndrome Diagnosis

As shown in Multimedia Appendix 1, a total of 93 features related to TCM and modern medicine were collected in this study, including 4 general information items, 19 TCM symptoms, 2 blood gas values, 10 viral pneumonia indicators, 27 biochemical indicators, 30 blood routine indicators, and 1 coagulation indicator. Among these, patients’ TCM symptoms were further quantified using a TCM symptom scoring scale for viral pneumonia (Multimedia Appendix 2).

All patients with viral pneumonia were independently diagnosed with either the TCM cold or hot syndrome by 2 TCM chief physicians. If both physicians provided the same diagnosis, the patient was enrolled. In cases of disagreement, a third investigator participated in the discussion to reach a consensus. The diagnostic criteria for syndrome differentiation were based on the textbooks *TCM Diagnostics* [25] and *Guiding Principles of Clinical Research on New Drugs of Traditional Chinese Medicine* [26]. These criteria include the 4 classical diagnostic methods used to determine TCM cold and hot syndrome patterns: inspection, auscultation, inquiry, and palpation, to accurately record the TCM characteristics.

### Model Training

**Figure 1.**
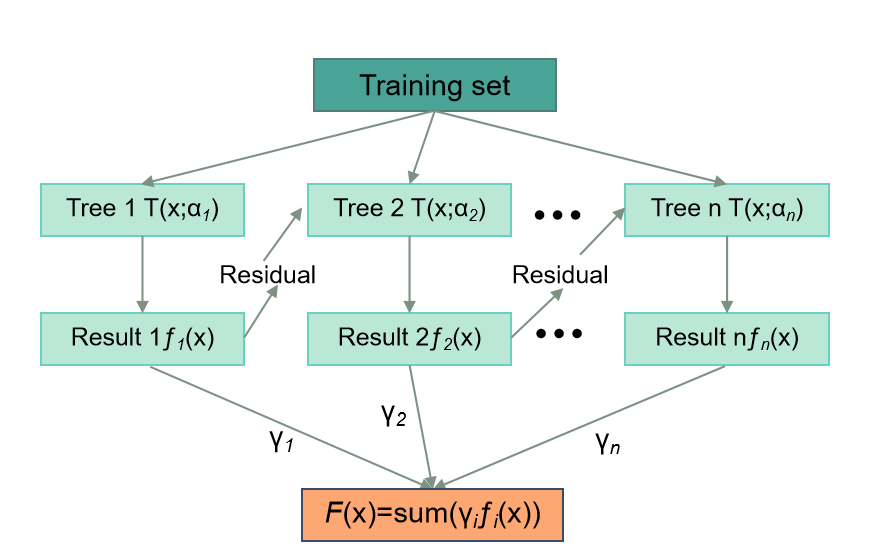
Gradient boosting machine decision tree composition and prediction flowchart.

In this study, 8 machine learning algorithms (GBM, LR, RF, XGB, LGB, RIDGE, LASSO, and SVM) were used to train a dataset containing all features, and GBM was selected as the final classification model. As shown in Figure 1, GBM operates on the principle of ensemble learning, constructing the final prediction model by iteratively training a series of weak learners, typically decision trees. The core mechanism of GBM lies in its ability to correct the prediction errors of previous decision trees. Each newly generated decision tree is trained to fit the residuals—the differences between the actual values and the predicted values—of the previous model, thereby continuously reducing the overall loss function. Specifically, GBM begins by constructing an initial decision tree based on the training data, and then calculates the prediction error of this tree. A second tree is built to model the residuals, with its predictions aimed at correcting the deficiencies of the first tree. This process continues iteratively until a preset number of iterations is reached or a stopping condition is met. Figure 1 illustrates the structure and decision-making process of multiple decision trees in the GBM model. Each decision tree branches based on input features, and the final prediction is obtained through a weighted sum of the predictions from all individual trees.

The number of top-ranking indexes was gradually increased, and the hyperparameters n_estimators and max_depth were tuned. The final model consisted of 13 top-ranking features, with n_estimators and max_depth set to 797 and 8, respectively. The GBM was implemented using the scikit-learn library (version 0.24.1) in Python (version 3.8.8; Python Foundation).

### Optimal Feature Ascertainment

“Feature importance,” “coef,” and “SHAP values” are 3 commonly used methods to assess the relationship between features and machine learning models, and they have received significant attention in recent research [27]. These methods calculate the relevance of each feature to the model’s predictions, which are then ranked in descending order to produce a feature importance ranking.

In tree-based models (eg, GBM, RF, XGB, LGB), feature importance is typically evaluated from 2 perspectives: split count and split gain. Split count refers to how often a particular feature is selected as a splitting point during tree construction; a higher count indicates greater importance. By contrast, split gain measures the improvement in the model’s objective function (eg, squared error loss) resulting from splitting on a given feature. A larger gain implies a greater contribution to model performance and hence higher importance. Additionally, SHAP (Shapley Additive Explanations) values offer a unified measure of feature importance based on game theory by calculating the marginal contribution of each feature to the model’s prediction. SHAP values provide both global and local interpretability of the model. In linear models (eg, RIDGE, LASSO, SVM, and LR), “coef” represents the coefficients assigned to each feature. These coefficients indicate the strength and direction of the linear relationship between each feature and the target variable. SHAP values can complement these coefficients by revealing nonlinear effects and feature interactions that may not be captured by standard linear models.

The insights gained from these methods, especially when combining traditional feature importance metrics with SHAP analysis, can aid researchers in uncovering hidden data patterns and insights, thereby supporting scientific investigations and informing practical applications. Accordingly, these methods are used to rank the feature importance for cold and hot syndromes of viral pneumonia and to ascertain the optimal features.

### Performance Evaluation and Model Validation

During the data preprocessing stage, a stratified sampling strategy was adopted to divide the original dataset, ensuring an 8:2 ratio between the training and validation sets. This approach preserved the proportional distribution of each class (cold and hot syndromes) as in the original dataset, thereby avoiding model evaluation bias due to imbalanced category distributions.

**Figure 2.**
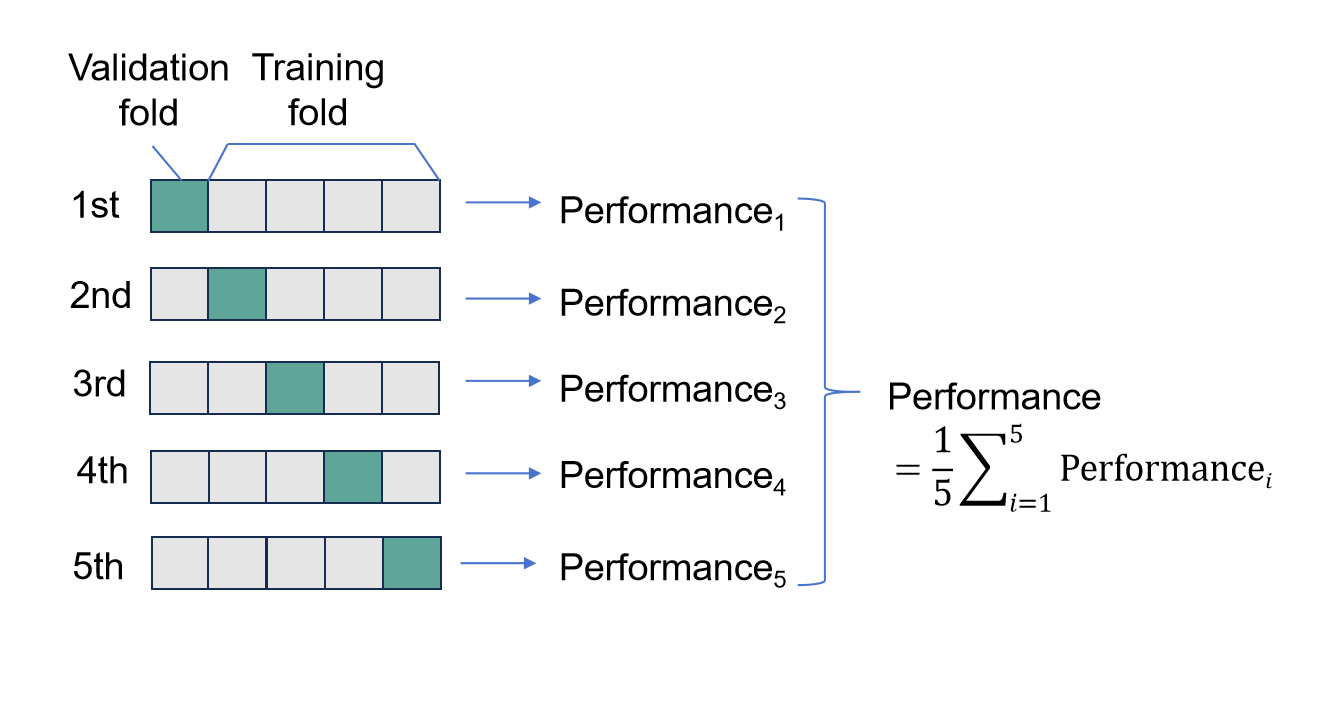
The 5-fold cross-validation process diagram.

Given the relatively small sample size, 5-fold cross-validation was applied to ensure model accuracy and reliability. As illustrated in Figure 2, the dataset was divided into 5 nonoverlapping subsets of equal size. In each round of validation, 1 subset was selected as the validation set (the blue portion in the figure), while the remaining 4 subsets constituted the training set (gray portions). This process was repeated for 5 rounds. In the first round, the first subset was the validation set, and the remaining 4 were used as the training set, resulting in Performance_₁_. In the second round, the second subset was used for validation, and so on, resulting in Performance_₂_ through Performance_5_. The final model performance was calculated as the average of these 5 evaluation results, with the formula $Performance\;=\;\sum_{i\;=\;1}^{5}Performance_{i}$. This strategy ensured that each sample was used for both training and validation, reduced the variance caused by random sample division, and improved the robustness and reliability of the model evaluation [28].

In addition, a comprehensive evaluation of the model was conducted using commonly used metrics, including sensitivity, specificity (SPE), accuracy (ACC), Matthews correlation coefficient (MCC), and area under the curve (AUC). These metrics are calculated based on the values of true positives (TPs), true negatives (TNs), false positives (FPs), and false negatives (FNs), as defined below:


(1) $$\text{Sens}=\frac{TP+FN}{TP}$$

(2) $$\text{Spec}=\frac{TN}{TN+FP}$$

(3) $$\text{ACC}=\frac{TP+TN}{TP+TN+FP+FN}$$

(4) $$\text{MCC}=\frac{TP\cdot TN-FP\cdot FN}{\sqrt{\left(TP+FP)(TP+FN)(TN+FP)(TN+FN\right)}}$$

The receiver operating characteristic curve, also known as the sensitivity curve, is a graphical representation of the performance of a classification model across various evaluation thresholds. It plots the true-positive rate against the false-positive rate at different threshold settings. The true-positive rate reflects the proportion of actual positive cases correctly identified by the model, while the false-positive rate represents the proportion of negative cases incorrectly classified as positive. The AUC provides a single summary metric of the classifier’s overall performance, with values ranging from 0 to 1. A higher AUC value indicates better classification performance. Therefore, AUC is widely used as a standard metric to evaluate the performance of classification models. The formula for calculating AUC is as follows:


(5) $$\text{AUC}=\int_{0}^{1}TPR(\text{FDR})\;d\text{FDR}$$

### Ethical Considerations

The study was approved by the Medical Ethics Review Committee of the Second People’s Hospital of Lanzhou City and Lanzhou Heavy Ion Hospital (approval number 2021-026-01). Informed consent was obtained on an opt-out basis, and all data were anonymized. No financial or material incentives were provided to participants.

### Statistical Analysis

All data were analyzed using SPSS version 27.0 (IBM Corp). Categorical variables are presented as counts and percentages, whereas continuous variables are reported as means and SDs or IQRs, as appropriate. Normality was assessed using the Kolmogorov-Smirnov test. Student 2-tailed *t* test was used to compare parametric continuous variables, while the Mann-Whitney *U* test was used for nonparametric variables, the *χ*^2^ test was applied for categorical variables, and the Fisher exact test was used for 2×2 contingency tables. Binomial distribution tests were performed for each feature within the cold syndrome and hot syndrome groups. No correction for multiple comparisons was applied. A 2-sided *P*<.05 was considered statistically significant.

## Results

### Data Extraction

**Figure 3.**
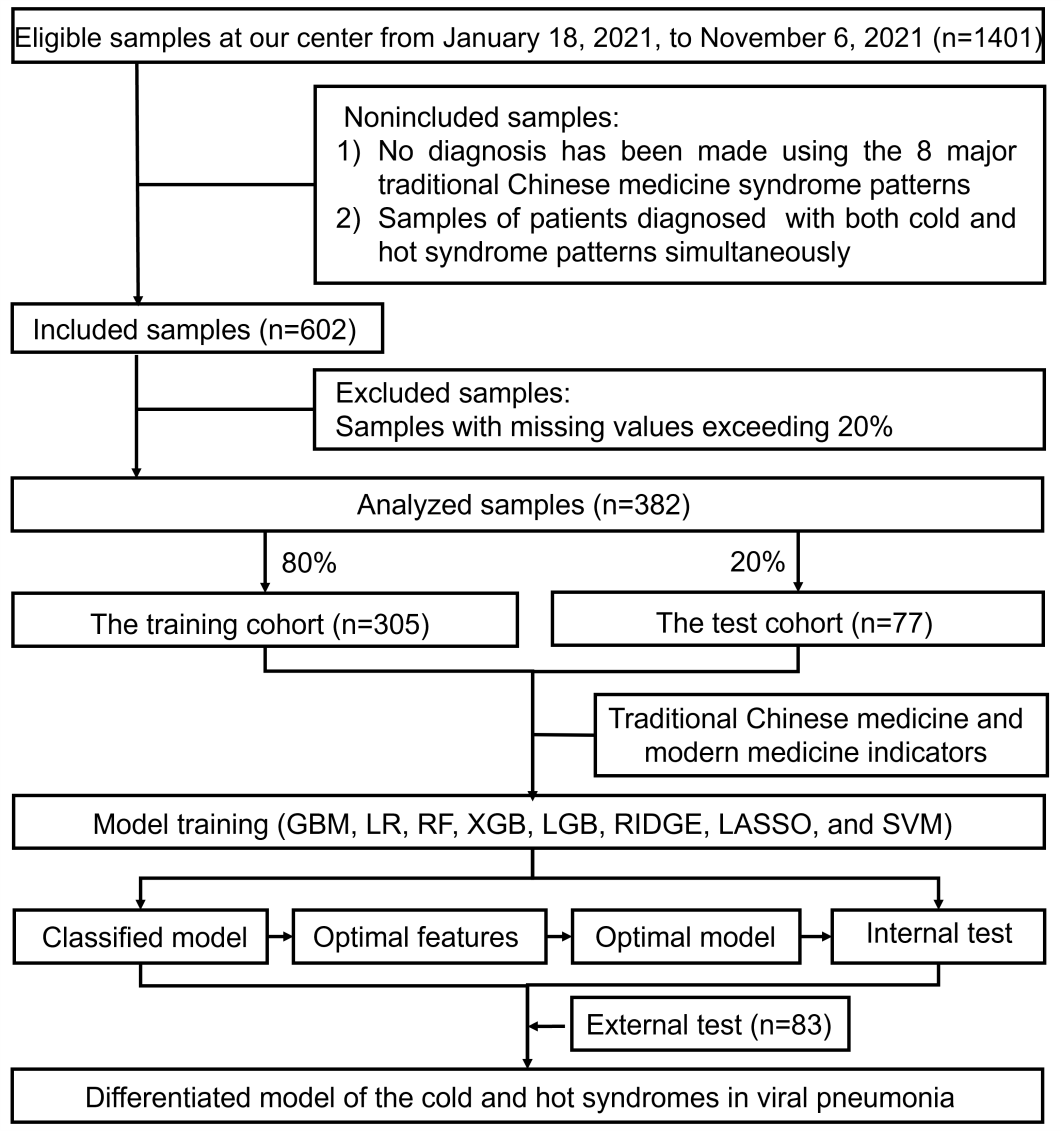
Research flow for data processing and predictive model construction. After preliminary screening of the collected data, 382 samples of patients with viral pneumonia with TCM cold and hot syndromes were obtained. The dataset was randomly divided into training and test sets in an 8:2 ratio. Traditional Chinese medicine and modern medical indicators were extracted through data processing and statistical analysis. The performance of the 8 machine learning models (gradient boosting machine [GBM], logistic regression [LR], random forest [RF], extreme gradient boosting [XGB], light gradient boosting machine [LGB], ridge regression, least absolute shrinkage and selection operator [LASSO], and support vector machine [SVM]) was evaluated. Based on a comprehensive evaluation of all models, the optimal classification algorithm and feature set were identified. The final model’s performance was further assessed using internal and external test cohorts.

We included only individual samples from patients with viral pneumonia and excluded those that were not diagnosed using the Eight Principle Syndromes (cold and hot, sthenia and asthenia, exterior and interior, and yin and yang), as well as those simultaneously diagnosed with cold and hot syndrome. A total of 602 eligible samples were retained. Samples with more than 20% (ie, 19/93 features) missing values were further excluded, and the remaining missing values were imputed using the median of each variable. Ultimately, 382 complete samples were included in the final analysis and used to construct the mathematical model. The dataset was split into training and testing sets in an 8:2 ratio. Subsequently, by integrating indicators from TCM and modern medicine and applying 8 machine learning algorithms, we identified the optimal model for differentiating TCM cold-hot syndromes in viral pneumonia through 5-fold cross-validation. The model’s performance was further assessed using an external validation cohort. The data processing workflow and model construction process are illustrated in Figure 3.

### Model Training Based on TCM and Modern Medicine Features

**Figure 4.**
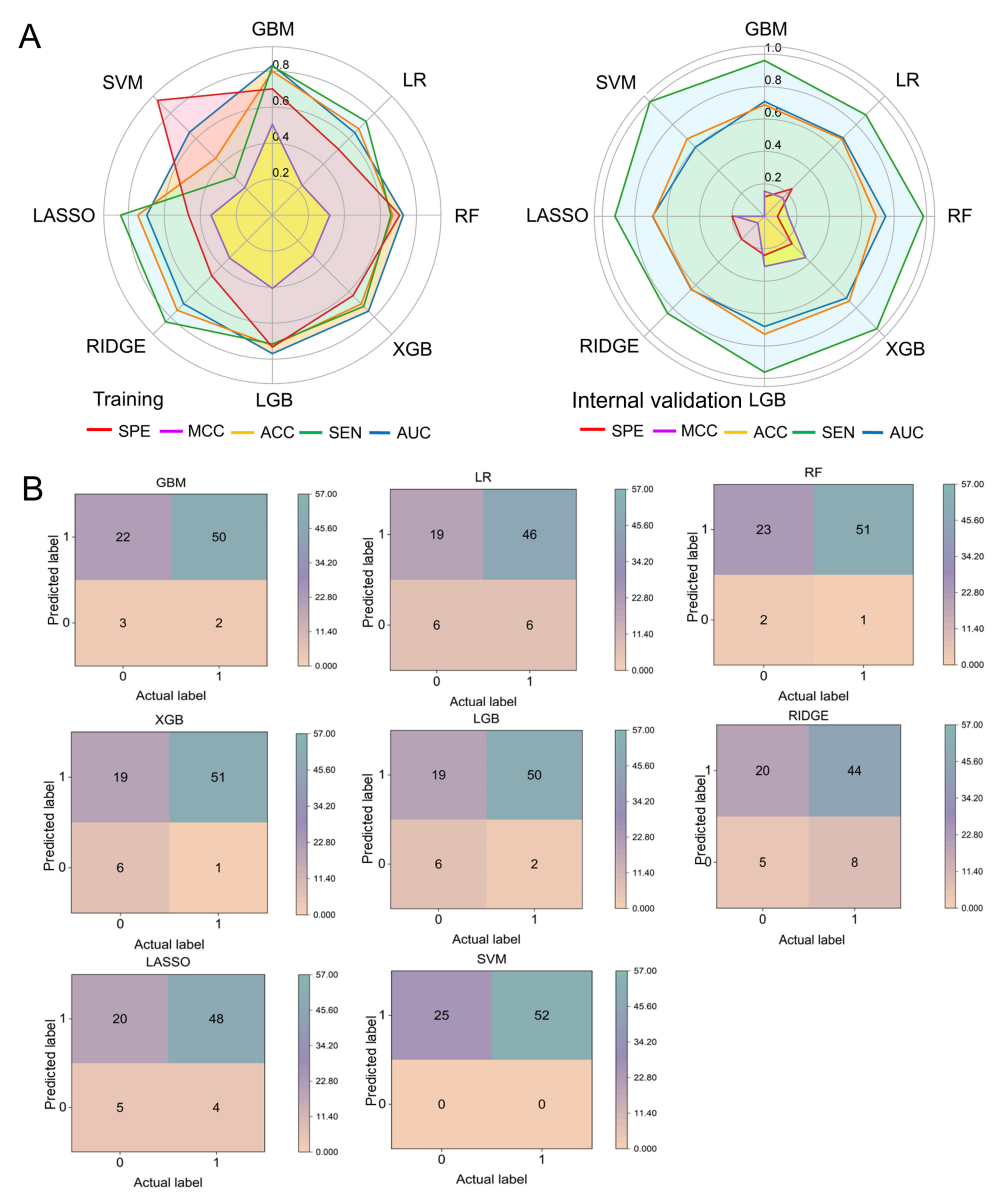
Performance evaluation of the cold and hot syndrome identification models based on integrated traditional Chinese medicine (TCM) and modern medical features. (A) Radar chart comparing the performance of 8 machine learning models in the training and internal validation cohorts. (B) Confusion matrix analysis of the 8 models in the internal validation cohort. ACC: accuracy; AUC: area under the curve; GBM: gradient boosting machine; LASSO: least absolute shrinkage and selection operator; LGB: light gradient boosting machine; LR: logistic regression; MCC: Matthews correlation coefficient; RF: random forest; RIDGE: ridge regression; SEN: sensitivity; SPE: specificity; SVM: support vector machine; XGB: extreme gradient boosting.

Modern medical indicators based on laboratory testing provide the possibility for intelligent discrimination of TCM syndromes. Therefore, in this study, modern medical indicators were integrated with TCM features for further exploration. A total of 93 features were used to construct the model. The evaluation metrics for models using all 93 features are presented in Table 1 and Figure 4A. We found that GBM and LGB achieved the highest AUC scores of 0.8329 and 0.7693, respectively, indicating their superior overall discriminative performance. GBM also obtained the highest ACC score of 0.8000, closely followed by RIDGE with a score of 0.7475, suggesting strong overall classification accuracy. LASSO, RIDGE, and GBM achieved the highest sensitivity scores of 0.8430, 0.8388, and 0.8283, respectively, indicating good performance in correctly identifying positive samples and reducing false negatives. SVM, LGB, RF, and GBM achieved the highest specificity scores, suggesting these models had the lowest false-positive rates. Both GBM and LGB also achieved relatively high MCC scores of 0.5043 and 0.4068, respectively, demonstrating their effectiveness in handling imbalanced datasets.

Internal validation showed that GBM, RF, and XGB achieved the highest AUC scores of 0.7085, 0.7477, and 0.7146, respectively. Additionally, we calculated the confusion matrix for each model in the internal validation cohort, as shown in Figure 4B. The sum of correctly predicted cold and hot samples reflects the overall predictive accuracy of each model. GBM, LR, RF, XGB, LGB, RIDGE, LASSO, and SVM correctly predicted 53, 52, 53, 57, 56, 49, 53, and 52 cold and hot samples, respectively. These findings further demonstrate the effectiveness of these models in distinguishing cold and heat patterns in viral pneumonia.

Overall, the results suggest that models incorporating both TCM and modern medicine features exhibit strong predictive performance for differentiating between cold and hot syndromes in viral pneumonia. Among them, the GBM algorithm demonstrated a particularly prominent discriminative capability. Therefore, GBM was selected as the optimal classified model based on the full set of 93 features for subsequent analysis.

**Table 1.** Comparison of models based on 5-fold cross-validation results using 93 features from traditional Chinese medicine and modern medicine.

Data cohort and evaluation	Gradient boosting machine	Logistic regression	Random forest	Extreme gradient boosting	Light gradient boosting machine	Ridge regression	Least absolute shrinkage and selection operator	Support vector machine
Training								
Area under the curve	0.8329	0.6477	0.7272	0.7546	0.7693	0.6970	0.6974	0.6514
Accuracy	0.8000	0.6787	0.6689	0.6984	0.7246	0.7475	0.7475	0.4459
Sensitivity	0.8283	0.7365	0.6582	0.7172	0.7158	0.8388	0.8430	0.2975
Specificity	0.7016	0.5187	0.7066	0.6326	0.7327	0.4764	0.4656	0.9018
Matthews correlation coefficient	0.5043	0.2341	0.3190	0.3177	0.4068	0.3364	0.3407	0.2161
Internal validation								
Area under the curve	0.7085	0.6854	0.7477	0.7146	0.6800	0.6392	0.6908	0.6031
Accuracy	0.6883	0.6753	0.6883	0.7403	0.7273	0.6364	0.6883	0.6753
Sensitivity	0.9615	0.8846	0.9808	0.9808	0.9615	0.8462	0.9231	1.0000
Specificity	0.1200	0.2400	0.0800	0.2400	0.2400	0.2000	0.2000	0.0000
Matthews correlation coefficient	0.1549	0.1609	0.1471	0.3596	0.3093	0.0577	0.1794	0.0000

### Optimal Feature Ascertainment

Based on the optimum classified model (GBM) and the ranking of “feature importance,” we identified 23 key features with importance values more than twice the median. These were temperature (T), red cell distribution width-SD (RDW-SD), creatinine (CREA), total bilirubin (TBIL), globulin (GLO), C-reactive protein (CRP), unconjugated bilirubin (IBIL), white blood cell (WBC), neutrophil percentage (NEU%), aspartate transaminase/alanine transaminase (AST/ALT), total cholesterol (TCHO), thrombocytocrit (PCT), oxygen saturation under load (SaO_2_), age, platelets, mean platelet volume, lactate dehydrogenase, mean corpuscular hemoglobin concentration, urea, erythrocyte/red blood cell, Na, ORF1ab gene of novel coronavirus by nose test (nCovORF1ab), and albumin.

**Figure 5.**
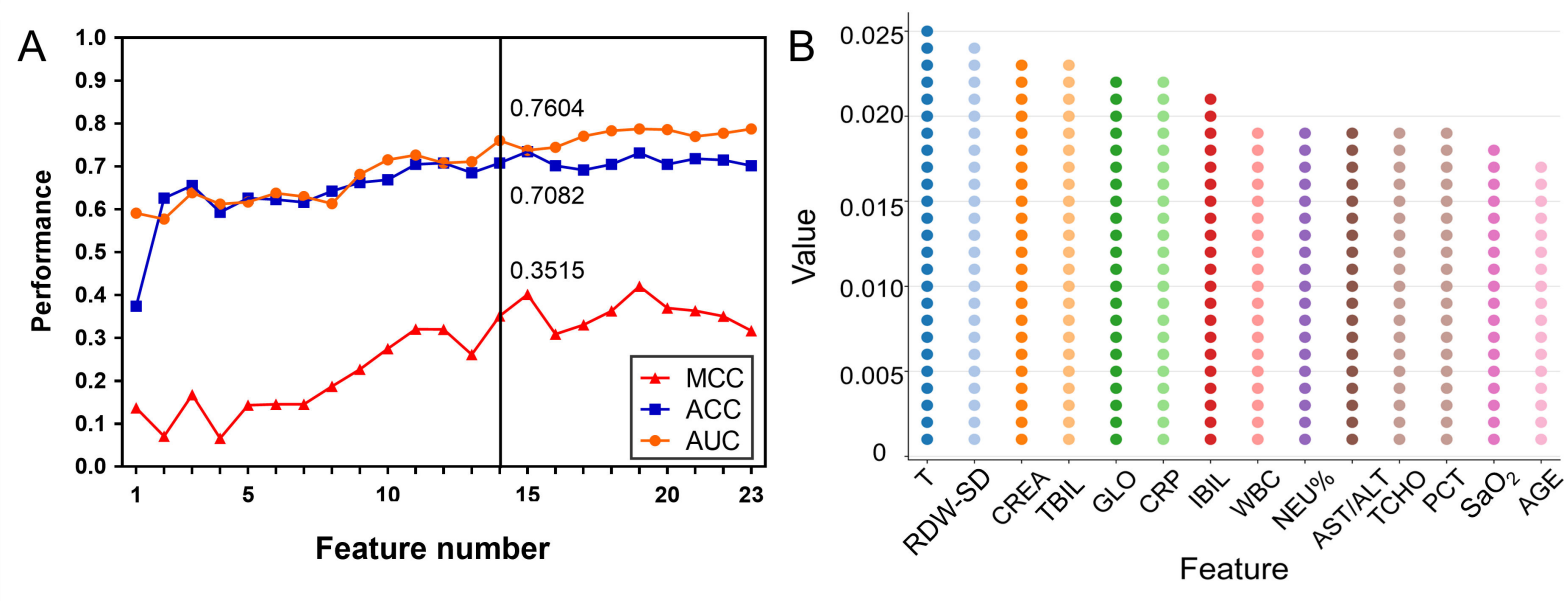
Identification of key features based on feature importance analysis and model cross-validation. (A) Effect of the number of selected key features (n=23) on model performance using the gradient boosting machine (GBM) algorithm. (B) Feature importance scores of the top 14 key features identified by the GBM model. ACC: accuracy; AUC: area under the curve; AST/ALT: aspartate transaminase/alanine transaminase; CREA: creatinine; CRP: C-reactive protein; GLO: globulin; IBIL: unconjugated bilirubin; MCC: Matthews correlation coefficient; NEU%: neutrophil percentage; PCT: thrombocytocrit; RDW-SD: red cell distribution width-SD; SaO_2_: oxygen saturation under load; T: temperature; TBIL: total bilirubin; TCHO: total cholesterol; WBC: white blood cell.

Subsequently, to identify the best-performing model with the fewest features, we examined how the number of top-ranked features influenced model performance (AUC, ACC, and MCC) using the GBM algorithm. As shown in Figure 5A and B, as the number of features increases, model performance gradually improves. Notably, at the 14-feature mark, the model achieved strong performance with an AUC of 0.7604, an ACC of 0.7082, and an MCC of 0.3515. To validate the robustness of our feature selection approach, we also used the SHAP feature importance ranking to select the top 14 features and construct a model. This model achieved an AUC of 0.7406, which was slightly lower than that obtained using the feature importance method (0.7604). Therefore, the feature importance method was ultimately retained as the feature selection approach for this study.

Finally, considering both the practicality of indicator testing and the feasibility of the clinical application, this study selected the top 14 features, excluding SaO_2_ (under load), as the optimal set for differentiating between cold and hot syndromes in viral pneumonia. These features are temperature (T), RDW-SD, CREA, TBIL, GLO, CRP, IBIL, WBC, NEU%, AST/ALT, TCHO, PCT, and age (AGE), as illustrated in (Multimedia Appendix 3).

### Performance Evaluation and Optimal Model Establishment

**Figure 6.**
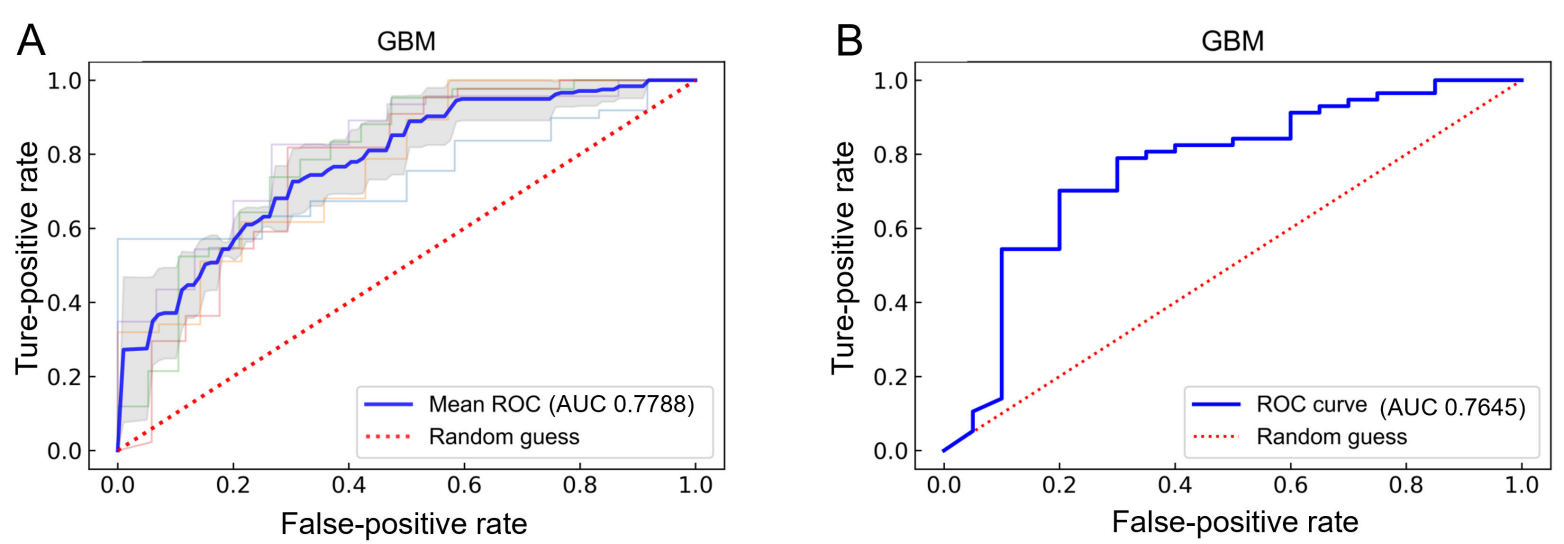
ROC curves and corresponding AUCs for cross-validation on the training and test sets. (A) ROC curve and classification performance of the final model set on the training set using 5-fold cross-validation. The x-axis represents the false-positive rate (FPR), and the y-axis represents the true-positive rate (TPR). (B) ROC curve and classification performance of the final model on the test set. The x-axis represents the FPR, and the y-axis represents the TPR. AUC: area under the curve; GBM: gradient boosting machine; ROC: receiver operating characteristic.

To achieve optimal GBM model performance, we reconstructed the GBM model based on 13 optimizing features. Ultimately, the model was adjusted to threshold=0.99999, n_estimators=797, max_depth=8, min_samples_split=2, and min_samples_leaf=1. Simultaneously, the performance of the model with 13 features was further evaluated using 5-fold cross-validation, and the results suggested that the model performance was outstanding, as the average AUC value reached 0.7788 (Figure 6A). Model verification results showed that the AUC was 0.7645 by the internal test cohort (Figure 6B). Furthermore, the additional external test results showed that the model performance for AUC, ACC, sensitivity, specificity, and MCC is 0.8428, 0.6627, 0.7222, 0.6170, and 0.3369, respectively. These results validated the excellent performance of the model.

**Figure 7.**
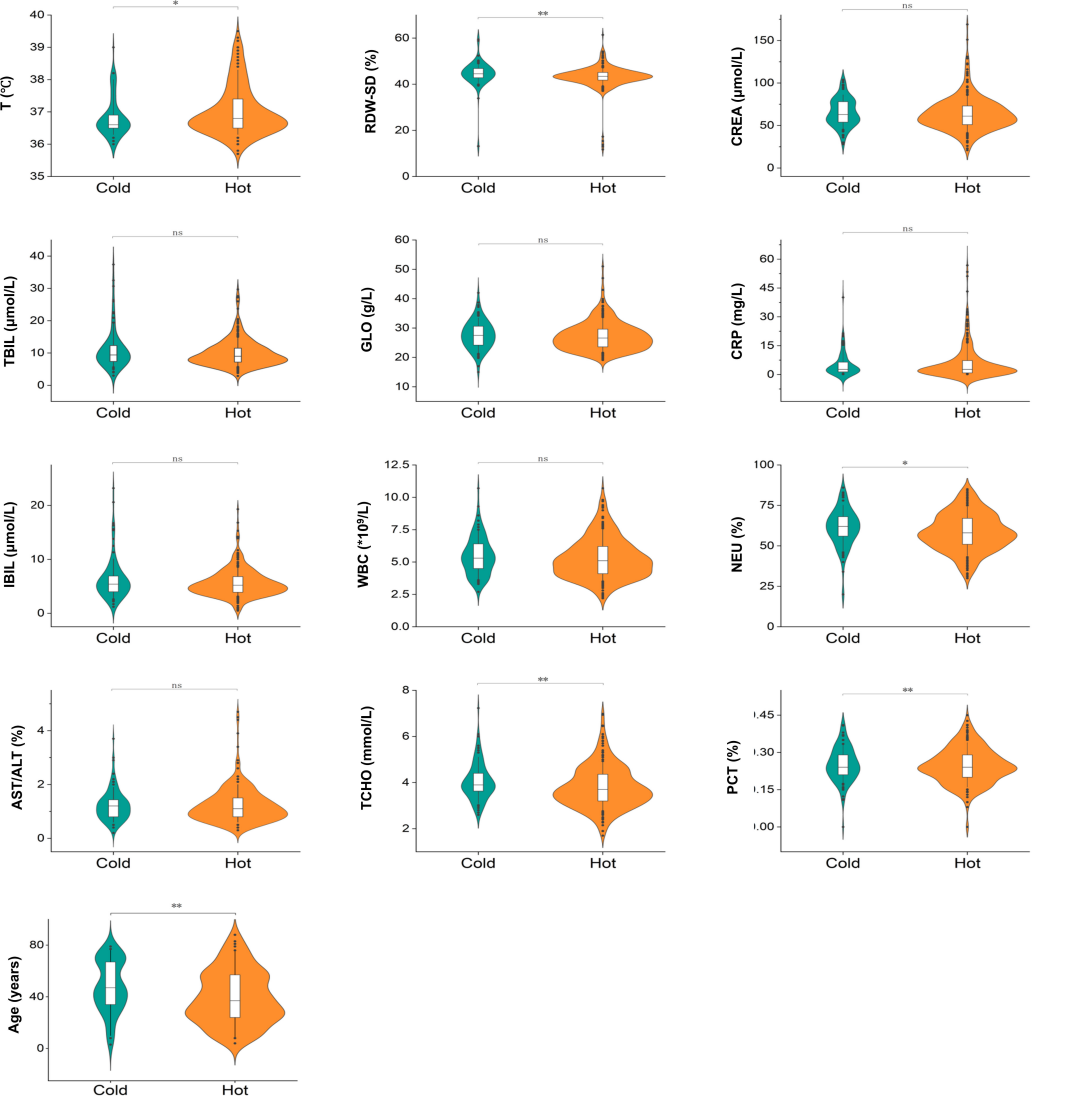
Violin plots showing the relationships between cold and hot syndromes and the expression levels of optimal clinical features. The horizontal axis represents the different syndromes (cold and hot), while the vertical axis indicates the expression levels of the corresponding clinical features. The plots illustrate the distribution and density of feature values, highlighting differences between the 2 syndrome groups. AST/ALT: aspartate transaminase/alanine transaminase; CREA: creatinine; CRP: C-reactive protein; GLO: globulin; IBIL: unconjugated bilirubin; NEU%: neutrophil percentage; ns: not significant; PCT: thrombocytocrit; RDW-SD: red cell distribution width-SD; T: temperature; TBIL: total bilirubin; TCHO: total cholesterol; WBC: white blood cell; *P<.05, **P<.01 versus the cold.

Moreover, we found that the temperature in the hot syndrome group was significantly higher than in the cold syndrome group (*P*=.02). By contrast, RDW-SD (*P*<.001), NEU% (*P*=.01), TCHO (*P*=.003), PCT (*P*<.001), and AGE (*P*<.001) were significantly lower in the hot syndrome group compared with the cold syndrome group, as shown in Table 2 and Figure 7. These results align with previous studies, which have reported that temperature, AGE, CREA, and RDW-SD are closely associated with the severity of viral pneumonia [29-33], supporting the validity of our findings.

These results suggest that machine learning algorithms perform well in differentiating between TCM cold and hot syndromes in viral pneumonia. The optimal model was constructed using 13 key features (T, RDW-SD, CREA, TBIL, GLO, CRP, IBIL, WBC, NEU%, AST/ALT, TCHO, PCT, and AGE), combined with the GBM algorithm. Among these, temperature (T), RDW-SD, NEU%, TCHO, PCT, and AGE appear to be the most relevant features for predicting cold and hot syndrome.

**Table 2.** The 13 optimal features for distinguishing between cold and hot syndromes in analysis.

Clinical features	Cold (n=97), median (IQR)	Hot (n=285), median (IQR)	*P* value
Temperature (°C)	36.600 (36.500-36.950)	36.800 (36.500-36.95)	.02
Red cell distribution width (%)	44.500 (42.800-46.850)	43.400 (41.80-45.100)	<.001
Creatinine (μmol/L)	63.000 (54.000-78.000)	61.000 (51.000-73.500)	.13
Total bilirubin (μmol/L)	9.400 (7.350-12.450)	9.000 (7.200-11.550)	.31
Globulin (g/L)	27.500 (24.150-30.750)	26.600 (23.600-29.600)	.18
C-reactive protein (mg/L)	2.670 (1.355-6.530)	2.670 (0.865-7.235)	.41
Unconjugated bilirubin (μmol/L)	5.400 (4.000-6.900)	5.200 (3.900-6.800)	.28
White blood cell (×10^9^/L)	5.300 (4.450-6.400)	5.100 (4.100-6.250)	.22
Neutrophil percentage (%)	62.000 (56.000-68.500)	58.000 (50.500-67.000)	.01
Aspartate transaminase/alanine transaminase (%)	1.200 (0.800-1.445)	1.100 (0.800-1.500)	.68
Total cholesterol (mmol/L)	3.900 (3.630-4.400)	3.700 (3.190-4.365)	.003
Thrombocytocrit (%)	0.240 (0.210-0.290)	0.240 (0.200-0.290)	<.001
Age (years)	47.000 (34.000‐67.000)	37.000 (24.000‐57.000)	<.001

### Interactive Web Server

**Figure 8.**
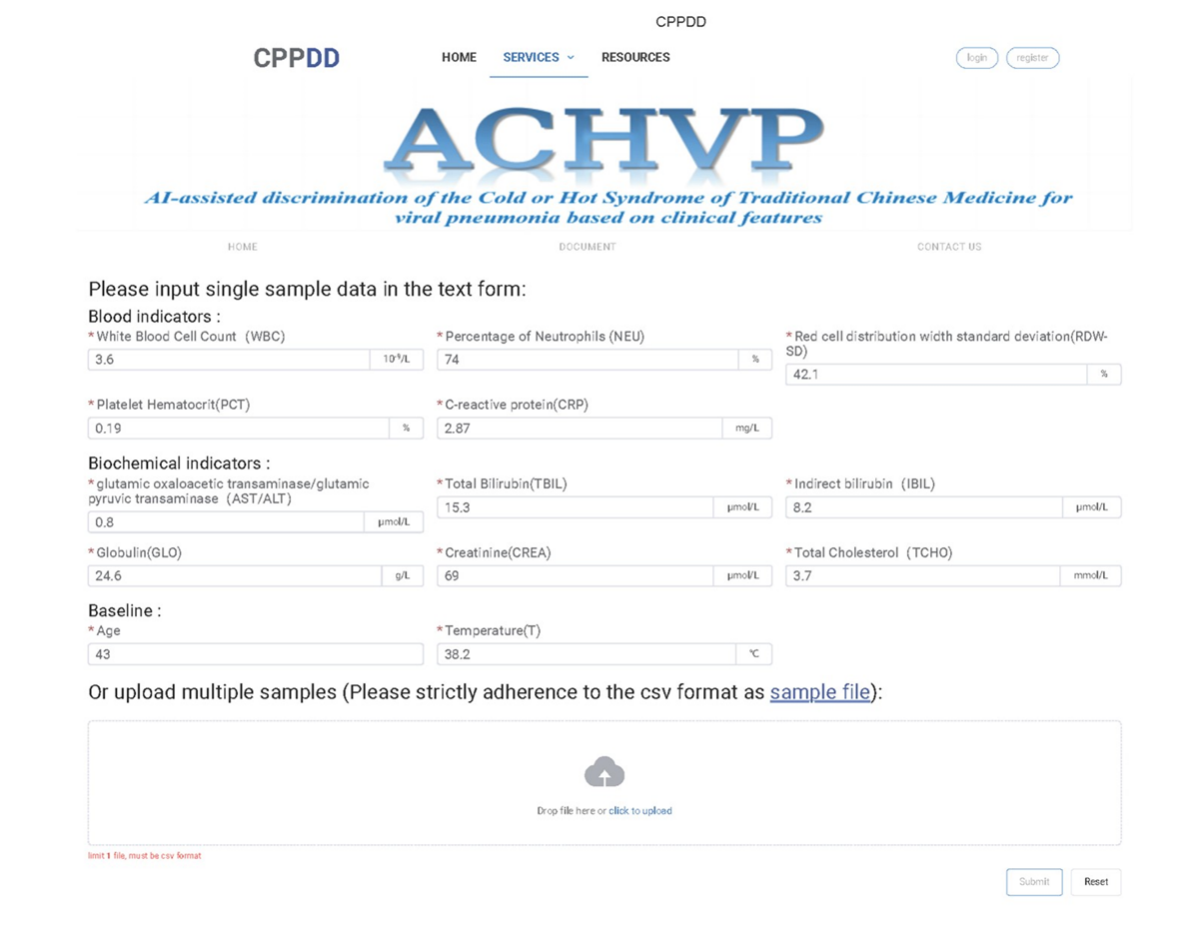
Web server of the ACHVP method.

To enhance user experience, an interactive web server named ACHVP was developed in this study, allowing users to test, explore, and experience the proposed method. The platform is designed to be highly convenient and user-friendly—users simply input relevant numerical data into designated textboxes and click the “Submit” button. The system then performs comprehensive data processing and analysis, presenting the predicted syndrome classification results on the output interface. The home page of the web server, developed for identifying cold and hot syndrome patterns in patients with viral pneumonia, is illustrated in Figure 8. The server is deployed in a Linux environment. The backend is developed in Python using the FastAPI framework to ensure efficient data processing and application programming interface management, while the front end is built with JavaScript and the Vue.js framework for a responsive and interactive user interface. Users can access the web server via any standard web browser, such as Google Chrome, Mozilla Firefox, or Safari, using the link specified in [34]. This web-based tool demonstrates significant potential as a practical diagnostic aid, facilitating early detection of cold and hot syndromes in patients with viral pneumonia. It can support TCM practitioners in syndrome differentiation and guide more precise Chinese herbal medicine prescriptions in clinical practice.

## Discussion

### Principal Findings

Dialectical treatment is a fundamental principle in TCM for diagnosing and treating diseases, with cold and hot syndromes serving as 2 key components for identifying the nature of illness [11,35-38]. In recent years, the study of TCM cold and hot syndrome has become a focal point in disease research. Numerous studies have shown that cold and hot syndromes also exhibit major TCM diagnostic patterns in various viral pulmonary diseases, including COVID-19 and SARS [8,14]. Based on these syndrome classifications, TCM has demonstrated a prominent role in alleviating symptoms, shortening treatment duration, and reducing the progression to severe pneumonia in patients with viral pneumonia [39-43]. For example, formulas such as Hanshiyi for cold syndrome [44] and Jinhua Qinggan Granules for hot syndrome have shown favorable clinical efficacy [45]. Therefore, the rapid, accurate, and specific identification of cold or hold syndrome is essential for the effective application of TCM in the treatment of viral pulmonary diseases. However, the abstract and complex nature of TCM syndrome differentiation poses challenges in accurately distinguishing between cold and hot syndromes in clinical practice. Machine learning offers a promising solution to this challenge by supporting clinicians in syndrome differentiation and facilitating more precise treatment decisions. Consequently, there is an urgent need to integrate TCM theories of cold and hot syndromes with modern laboratory diagnostics and machine learning approaches to advance the modernized differentiation and treatment of viral pneumonia.

In this study, 8 machine learning algorithms were applied to develop and evaluate a model for differentiating TCM cold and hot syndromes in viral pneumonia, using clinical data from 1484 patients across 2 medical centers. Among the models incorporating both TCM and modern medical features, the GBM model demonstrated superior learning capability, achieving an AUC of 0.8329. This indicates that GBM was particularly effective in distinguishing cold and hot syndromes based on a given dataset. The “feature importance” method of the GBM model was subsequently used to rank all features, from which the top 13 features were selected based on the highest AUC value. To validate the model’s effectiveness and generalizability, both internal and external test sets were used, each demonstrating strong discriminatory performance. As a result, the optimal model was constructed using the GBM algorithm and 13 selected features (T, RDW-SD, CREA, TBIL, GLO, CRP, IBIL, WBC, NEU%, AST/ALT, TCHO, PCT, and AGE), achieving an AUC of 0.7788. These findings suggest that effectively combining modern medical indicators, primarily based on laboratory tests, with TCM features primarily based on clinical observation may offer a viable pathway toward the modernization of TCM syndrome differentiation. This approach provides a foundation for the objectification and standardization of TCM diagnostic practices. Furthermore, the proposed model holds promise as a practical tool to assist clinicians in accurately identifying cold and hot syndromes in viral pneumonia, thereby informing appropriate and individualized treatment strategies.

### Comparison With Prior Work

Machine learning, as an advanced artificial intelligence technology, has demonstrated broad potential in the field of “artificial intelligence+TCM diagnoses.” For example, Zhang et al [46] proposed a comprehensive model using convolutional neural networks to classify 187 types of TCM diseases. Wen et al [47] also applied deep neural networks to identify tongue image data and determine patients’ constitutions, effectively addressing the imbalance problem among constitution categories. However, limited research has focused on identifying TCM cold and hot syndromes in viral pneumonia by integrating both TCM theory and modern medicine through machine learning approaches. Although some scholars have attempted to explore the cold and hot attributes of diseases to guide clinical treatment, most studies have been limited to network analysis, clustering, or laboratory testing, without incorporating artificial intelligence methods. For instance, Li et al’s [11] network analysis revealed that the leptin biomarker indicated low levels of energy metabolism in patients with cold syndrome, while the human monocyte chemoattractant protein-1 (CCL2/MCP1) biomarker suggested intensified immune regulation in patients with hot syndrome, based on a study involving patients with chronic superficial gastritis and chronic atrophic gastritis. Wang et al [13] differentiated heat and cold patterns in rheumatoid arthritis through cluster and factor analyses to guide clinical medication. Wu et al [48] investigated TCM cold and hot constitutions using pulse wave parameters such as augmentation index and heart rate variability. Although some individual studies have applied artificial intelligence to differentiate cold and hot properties, most efforts have been limited to identifying the properties of Chinese herbal medicines. For example, Lin et al [49] developed a classification strategy for the cold and hot properties of Chinese herbal medicines using artificial intelligence and biological experiments. Wei et al [50] applied machine learning techniques to intelligently identify the cold-hot nature of Chinese herbal medicines based on the similarity of their volatile oil ingredients. Therefore, this study innovatively constructs a model for differentiating cold and hot syndromes in viral pneumonia using machine learning approaches that integrate TCM and modern medicine, aiming to assist in diagnosis and medication guidance for viral pneumonia in clinical practice.

It is worth noting that in this study, we collected a large number of clinical features, including patients’ symptoms, signs, laboratory examination results, and other relevant information, which were essential for training and testing the machine learning models. As shown in Table 3, based on the model training results using only 19 TCM features, we found that the AUC values of GBM, LR, RF, XGB, LGB, RIDGE, LASSO, and SVM were 0.6942, 0.6317, 0.6783, 0.6787, 0.6055, 0.6151, 0.6145, and 0.5874, respectively. Meanwhile, the study also attempted to build models using only 70 laboratory results. The AUC values for GBM, LR, RF, XGB, LGB, RIDGE, LASSO, and SVM were 0.6799, 0.6282, 0.6797, 0.6910, 0.7345, 0.6637, 0.6157, and 0.6618, respectively. Among them, the GBM model constructed using TCM features achieved an AUC of 0.6942, outperforming the GBM model constructed using laboratory features, which had an AUC of 0.6799. Internal validation and the confusion matrix results also demonstrated performance consistent with the training results, as shown in Multimedia Appendix 4. These findings suggest that the models possess auxiliary diagnostic value for differentiating TCM cold and hot syndromes in viral pneumonia.

**Table 3.** Comparison of model results by 5-fold cross-validation based on different features of traditional Chinese medicine and modern medicine.

Features and evaluation	Gradient boosting machine	Logistic regression	Random forest	Extreme gradient boosting	Light gradient boosting machine	Ridge regression	Least absolute shrinkage and selection operator	Support vector machine
Traditional Chinese medicine								
Area under the curve	0.6942	0.6317	0.6783	0.6787	0.6055	0.6151	0.6145	0.5874
Accuracy	0.6851	0.5312	0.5509	0.6031	0.5155	0.5180	0.5049	0.7508
Sensitivity	0.7365	0.4737	0.5077	0.5908	0.4389	0.4258	0.4117	0.9928
Specificity	0.5804	0.7316	0.7457	0.7006	0.7233	0.7854	0.7723	0.2601
Matthews correlation coefficient	0.2737	0.1689	0.2145	0.2400	0.1332	0.1821	0.1563	0.2385
Modern medicine								
Area under the curve	0.6799	0.6282	0.6797	0.6910	0.7345	0.6637	0.6157	0.6618
Accuracy	0.7508	0.7508	0.7541	0.7672	0.7803	0.7639	0.7180	0.7639
Sensitivity	0.9441	0.9185	0.9742	0.9400	0.9314	0.8968	0.8539	1.0000
Specificity	0.1257	0.2105	0.0410	0.2067	0.2914	0.3324	0.2762	0.0000
Matthews correlation coefficient	0.1269	0.1585	0.0335	0.1927	0.2871	0.2672	0.1752	0.0000
Traditional Chinese medicine and modern medicine								
Area under the curve	0.8329	0.6477	0.7272	0.7546	0.7693	0.6970	0.6974	0.6514
Accuracy	0.8000	0.6787	0.6689	0.6984	0.7246	0.7475	0.7475	0.4459
Sensitivity	0.8283	0.7365	0.6582	0.7172	0.7158	0.8388	0.8430	0.2975
Specificity	0.7016	0.5187	0.7066	0.6326	0.7327	0.4764	0.4656	0.9018
Matthews correlation coefficient	0.5043	0.2341	0.3190	0.3177	0.4068	0.3364	0.3407	0.2161

Based on “feature importance” or “coef,” the 8 models were further used to evaluate the significance of TCM features in distinguishing cold and hot syndromes of viral pneumonia. We found that, according to the significance values, there were 7 common features with importance values equal to or greater than the median in the GBM, RF, XGB, and LGB models, namely, fatigue, sore throat, fever, expectoration, cough, body pain, and diminished sense of smell. Interestingly, sore throat and fever also appeared among the features with importance values equal to or greater than the median in LR, RIDGE, LASSO, and SVM models. Based on this, we believe that fatigue, sore throat, fever, expectoration, cough, body pain, and diminished sense of smell may be the primary distinguishing factors for differentiating between cold and hot syndromes in the TCM diagnosis of viral pneumonia, which aligns with the key symptomatology commonly used by clinical TCM practitioners [51,52]. Therefore, more attention should be given to these features, especially sore throat and fever, in the discrimination of cold and hot syndromes in viral pneumonia, as shown in Multimedia Appendix 5. Unfortunately, the AUC values of these models did not exceed 0.7. We boldly speculate that this might be due to the quantification of TCM features not aligning with modern medical standards. Specifically, TCM features lack precise numerical values and are typically categorized as none, mild, moderate, or severe. Moreover, the identification of TCM features primarily relies on the subjective judgment of TCM practitioners and years of clinical experience, leading to a lack of standardization and objectivity. These factors may introduce noise and reduce the effectiveness of model training. Therefore, it is essential to develop objective indicators for TCM syndrome differentiation, further supporting the original intent of our study. In subsequent experiments, we integrated modern medicine features with TCM indicators and were pleasantly surprised to find that, compared with models trained solely on TCM features, those based on integrated TCM and modern medical features demonstrated superior performance in distinguishing cold and hot syndromes in viral pneumonia across 8 machine learning algorithms. This suggests that modern medical features can enhance the predictive value of TCM features.

In addition, the best model we developed indicated that the identification of cold and hot syndromes in viral pneumonia is closely related to 13 features: temperature, RDW-SD, CREA, TBIL, GLO, CRP, IBIL, WBC, NEU%, AST/ALT, TCHO, PCT, and AGE. Numerous studies have reported that temperature (T), CRP, and WBC hold statistical significance in distinguishing TCM cold and hot syndromes in rheumatoid arthritis [53]. Furthermore, features such as AGE, NEU%, RDW-SD, and CRP are strongly associated with the severity of viral pneumonia and the development of chronic lung sequelae. For example, a study by Liu et al [33] suggested that AGE and NEU% could serve as predictive factors for the severity of viral pneumonia. Shen et al [31] also reported that RDW-SD is a key indicator for assessing the severity of viral pneumonia. Additionally, CRP and AGE play crucial roles in the diagnosis of viral pneumonia [54,55], which is consistent with the findings of this study.

### Limitations

The accuracy of traditional TCM diagnosis of cold and hot syndromes largely depends on the skills and years of clinical experience of the practitioner. Subjective factors play a decisive role in this process. Compared with traditional TCM methods, the model developed in this study offers a more objective approach to differentiating cold and hot syndromes in viral pneumonia. It provides valuable decision support and medication guidance in clinical practice, especially for practitioners with limited TCM experience. This shift toward an objective, data-driven approach helps reduce reliance on subjective judgment, improves diagnostic accuracy, and ensures greater consistency in clinical decision-making. Additionally, the model can serve as a practical tool for TCM practitioners, especially in complex cases where traditional diagnostic methods may fall short.

Of course, this study has some limitations. First, it was a retrospective study, and all patient history data were obtained from the internal electronic medical record systems of the Second People’s Hospital of Lanzhou and Lanzhou Heavy Ion Hospital. This may have introduced inaccuracies in the collection and recording of medical history, making it difficult to avoid information bias. Moreover, due to the limited clinical sample size, the conclusions, although statistically sound, require further validation and refinement in real-world applications to enhance the model’s accuracy and stability.

In recent years, emerging large language models (LLMs) have demonstrated remarkable capabilities in natural language processing, knowledge integration, and complex reasoning [56]. Given the nature of TCM theory, which often involves complex textual descriptions and implicit knowledge, LLMs could potentially offer new perspectives for model development in this study. These models may better understand and integrate the rich, diverse information contained in TCM literature, clinical records, and expert knowledge, thereby enriching the feature set and enhancing the interpretability of the models. For instance, LLMs could analyze the semantic relationships among TCM symptoms, syndromes, and treatment methods, helping to extract more meaningful features that may be difficult to identify using traditional machine learning approaches. Moreover, LLMs could assist in standardizing and structuring the subjective and qualitative information in TCM, thereby reducing the impact of inconsistent data representation. However, applying LLMs also poses challenges, such as ensuring the reliability of the extracted knowledge, addressing potential biases inherent in pretrained models, and efficiently integrating LLM-derived features with existing laboratory test data. In future work, we plan to collect more data to establish a local database and explore the integration of LLMs with traditional machine learning algorithms. By fully leveraging the advantages of both approaches, we aim to further enhance the performance of the TCM cold and hot syndrome differentiation model and advance the modernization and standardization of TCM diagnosis.

### Conclusion

This study aimed to establish a differentiation model for TCM cold and hot syndromes in patients with viral pneumonia using machine learning by exploring correlations between cold/hot syndromes and features from both TCM and modern medicine (such as general information, TCM characteristics, blood gas values, viral pneumonia indicators, biochemical indicators, routine blood test indicators, and coagulation indicators). We found that machine learning algorithms based on integrated TCM and modern medicine features outperformed models that used only TCM or only modern medicine features. Specifically, a model composed of 13 optimal features (T, RDW-SD, CREA, TBIL, GLO, CRP, IBIL, WBC, NEU%, AST/ALT, TCHO, PCT, and AGE) and the GBM algorithm effectively differentiated TCM syndromes. This study is the first to integrate the TCM cold and hot syndrome theory with laboratory-based modern medical tests using machine learning. By establishing the relationship between TCM syndrome theory (cold and hot syndromes) and indicators from both TCM and modern medicine, we aim to provide a new auxiliary diagnostic method for the clinical differentiation of cold and hot syndromes. This can support practitioners in making comprehensive diagnoses and identifying effective Chinese herbal medicine treatments, while also offering new insights for the modernization and scientific interpretation of TCM syndrome differentiation theory to guide treatment.

## Supplementary material

10.2196/64725 Multimedia Appendix 1 All clinical features information of patients with viral pneumonia.

10.2196/64725 Multimedia Appendix 2 The traditional Chinese medicine symptom scoring scale for patients with viral pneumonia.

10.2196/64725 Multimedia Appendix 3 The optimal features for the TCM cold and hot syndrome discrimination of viral pneumonia. AGE: age; AST/ALT: aspartate transaminase/alanine transaminase; CREA: creatinine; CRP: C-reactive protein; GLO: globulin; IBIL: unconjugated bilirubin; NEU%: neutrophil percentage; PCT: thrombocytocrit; RDW-SD: red cell distribution width-SD; T: temperature; TBIL: total bilirubin; TCHO: total cholesterol; WBC: white blood cell.

10.2196/64725 Multimedia Appendix 4 The performance evaluation of the cold and hot syndrome identification model based on traditional Chinese medicine (TCM) features. (A) The performance evaluation’s radar chart of the 8 models based on TCM features. (B) The confusion matrix analysis of the 8 models based on TCM features.

10.2196/64725 Multimedia Appendix 5 Heat diagram of traditional Chinese medicine features on 8 screened models (the values of gradient boosting machine, random forest, extreme gradient boosting, and light gradient boosting were obtained using feature importance, and those of ridge regression, least absolute shrinkage and selection operator, support vector machine, and logistic regression were obtained using coef).

## Abbreviations

ACC: accuracy
ALT: alanine transaminase
AST: aspartate transaminase
AUC: area under the curve
CREA: creatinine
CRP: C-reactive protein
FN: false negative
FP: false positive
GBM: gradient boosting machine
GLO: globulin
IBIL: unconjugated bilirubin
LASSO: least absolute shrinkage and selection operator
LGB: light gradient boosting machine
LLM: large language model
LR: logistic regression
MCC: Matthews correlation coefficient
nCovORF1ab: Na, ORF1ab gene of novel coronavirus by nose test
NEU%: neutrophil percentage
PCT: thrombocytocrit
RDW: red cell distribution width
RF: random forest
RIDGE: ridge regression
SaO_2_: oxygen saturation under load
SEN: sensitivity
SHAP: Shapley additive explanations
SPE: specificity
SVM: support vector machine
T: temperature
TBIL: total bilirubin
TCHO: total cholesterol
TCM: traditional Chinese medicine
TN: true negative
TP: true positive
WBC: white blood cell
XGB: extreme gradient boosting
XGB: extreme gradient boosting
