# Supplementary material for: A Machine Learning Approach to Differentiate Cold and Hot Syndrome in Viral Pneumonia Integrating Traditional Chinese Medicine and Modern Medicine: Machine Learning Model Development and Validation
Source: JMIR Med Inform. 2025 Jul 16;13:e64725. doi: 10.2196/64725 (PMC12286567; doi:10.2196/64725)
Supplement: Multimedia Appendix 1 [file medinform-v13-e64725-s001.docx]

**Multimedia Appendix 1.** All clinical features information of viral pneumonia patients.

| Variable quantity | Number | Feature |
| --- | --- | --- |
| General information | 4 | sex, age, blood type, Temperature |
| TCM symptoms | 19 | fever, aversion to cold, sweat, headache, body pain, nasal congestion, runny nose, dry mouth, sore throat, diminished sense of smell, diminished sense of taste, cough, expectoration, chest tightness, shortness of breath, fatigue, anorexia, diarrhoea, constipation |
| Blood gas values | 2 | SaO_2_, SaO_2_ (under load) |
| Viral pneumonia indicators | 10 | IgM antibody titers, IgM antibody, IgG antibody titers, IgG antibody, nCovORF1ab (nose), nCoV-NP (nose), nCovORF1ab (oropharyngeal), nCoV-NP (oropharyngeal), COVID-19 nucleic acid test (nose), COVID-19 nucleic acid test (oropharyngeal) |
| Biochemical indicators | 27 | TBLL, UREG, CREA, DBIL, IBIL, TP, ALB, GLO, A.G, GLU, UA, TG, TCHO, HDL.C, LDL.C, MCK.MB, CK, ALT, AST, ALT, LDH, HBDH, K, Na, Cl, HCO3, AMY |
| Blood routine indicators | 30 | WBC, NEU, LYM, MON, EOS, BAS, NEU%, LYM%, MON%, EOS%, BAS%, HGB, RBC, HCT, MCV, MCH, MCHC, RDW-CV, RDW-SD, PLT, MPV, PCT, PDW, NRBC, NRBC%, P.LCR, CRP, Procalcitonin, SAA, IL-6 |
| Coagulation indicators | 1 | D-D |
