# Supplementary material for: A Machine Learning Approach to Differentiate Cold and Hot Syndrome in Viral Pneumonia Integrating Traditional Chinese Medicine and Modern Medicine: Machine Learning Model Development and Validation
Source: JMIR Med Inform. 2025 Jul 16;13:e64725. doi: 10.2196/64725 (PMC12286567; doi:10.2196/64725)
Supplement: Multimedia Appendix 2 [file medinform-v13-e64725-s002.docx]

**Multimedia Appendix 2.** The TCM symptom scoring scale for patients with viral pneumonia.

| TCM symptoms | None (0) | Mild (1) | Moderate (2) | Severe (3) |
| --- | --- | --- | --- | --- |
| fever | <37.3 | 37.2-37.9 | 38-38.5 | >38.5 |
| aversion to cold | none | aversion to cold without needing extra clothing | aversion to cold with needing extra clothing | aversion to cold with needing lots of clothing |
| sweat | none | sight sweat | sweat | excessive sweat |
| headache | none | mild and intermittent headache | severe and continuous headache | severe headache leading to inability to continue working |
| body pain | none | sight body pain | body pain | body pain, difficulty in bending and stretching |
| nasal congestion | none | feeling of congestion, dense nasal voice | sporadic nasal congestion | frequent nasal congestion |
| runny nose | none | occasionally | often | frequently |
| dry mouth | none | occasionally | often | frequently |
| sore throat | none | dry throat, slight pain | sore throat | severe sore throat |
| diminished sense of smell | none | diminished | obviously diminished | no sense of smell |
| diminished sense of taste | none | diminished | obviously diminished | no sense of taste |
| cough | none | occasionally | often | frequently |
| expectoration | <10ml  (day and night) | 10-50ml  (day and night) | 50-100ml  (day and night) | >100ml  (day and night) |
| chest tightness | none | mild chest tightness | obvious chest tightness | severe chest tightness |
| shortness of breath | none | shortness of breath with increased activity | shortness of breath with activity | shortness of breath without activity |
| fatigue | none | the body feels slightly fatigued but able to sustain light physical work | weakness in the limbs, barely able to sustain daily activities | feeling weak all over, unwilling to move all day |
| anorexia | none | reduce food intake by 1/4 | reduce food intake by 1/3 | reduce food intake by 1/2 |
| diarrhoea | none | hard stools, once every 1-2 days | hard stools, difficult bowel movement, once every 3-5 days | hard stools, difficult bowel movement, once every 5 days or more |
| constipation | none | unformed stool | loose stool, two to three times a day | loose stool, four or more times a day |
