# Supplementary figures and images for: A Machine Learning Approach to Differentiate Cold and Hot Syndrome in Viral Pneumonia Integrating Traditional Chinese Medicine and Modern Medicine: Machine Learning Model Development and Validation
Source: JMIR Med Inform. 2025 Jul 16;13:e64725. doi: 10.2196/64725 (PMC12286567; doi:10.2196/64725)

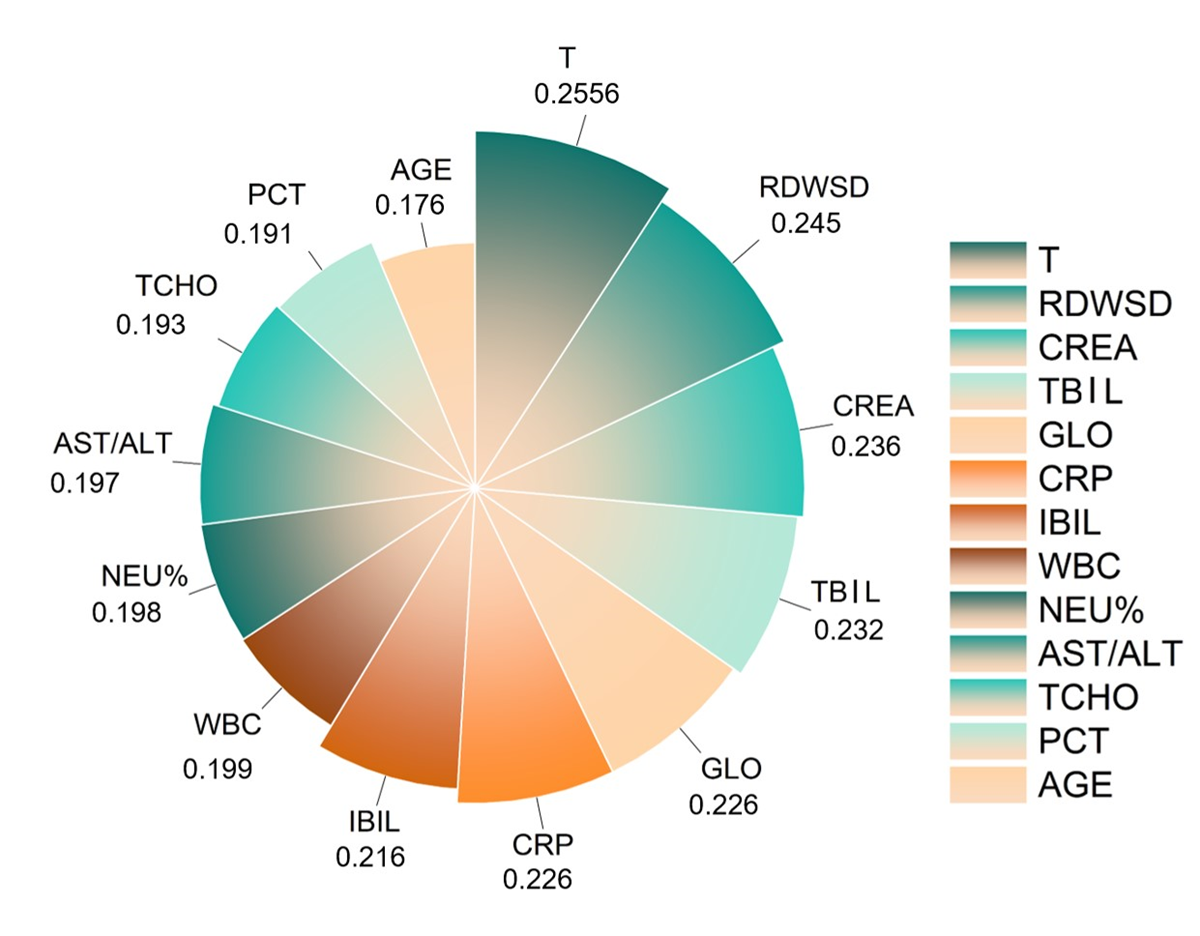

Supplement: Multimedia Appendix 3 [file medinform-v13-e64725-s003.png]
