# Supplementary material for: A Machine Learning Approach to Differentiate Cold and Hot Syndrome in Viral Pneumonia Integrating Traditional Chinese Medicine and Modern Medicine: Machine Learning Model Development and Validation
Source: JMIR Med Inform. 2025 Jul 16;13:e64725. doi: 10.2196/64725 (PMC12286567; doi:10.2196/64725)
Supplement: Multimedia Appendix 4 [file medinform-v13-e64725-s004.docx]

**
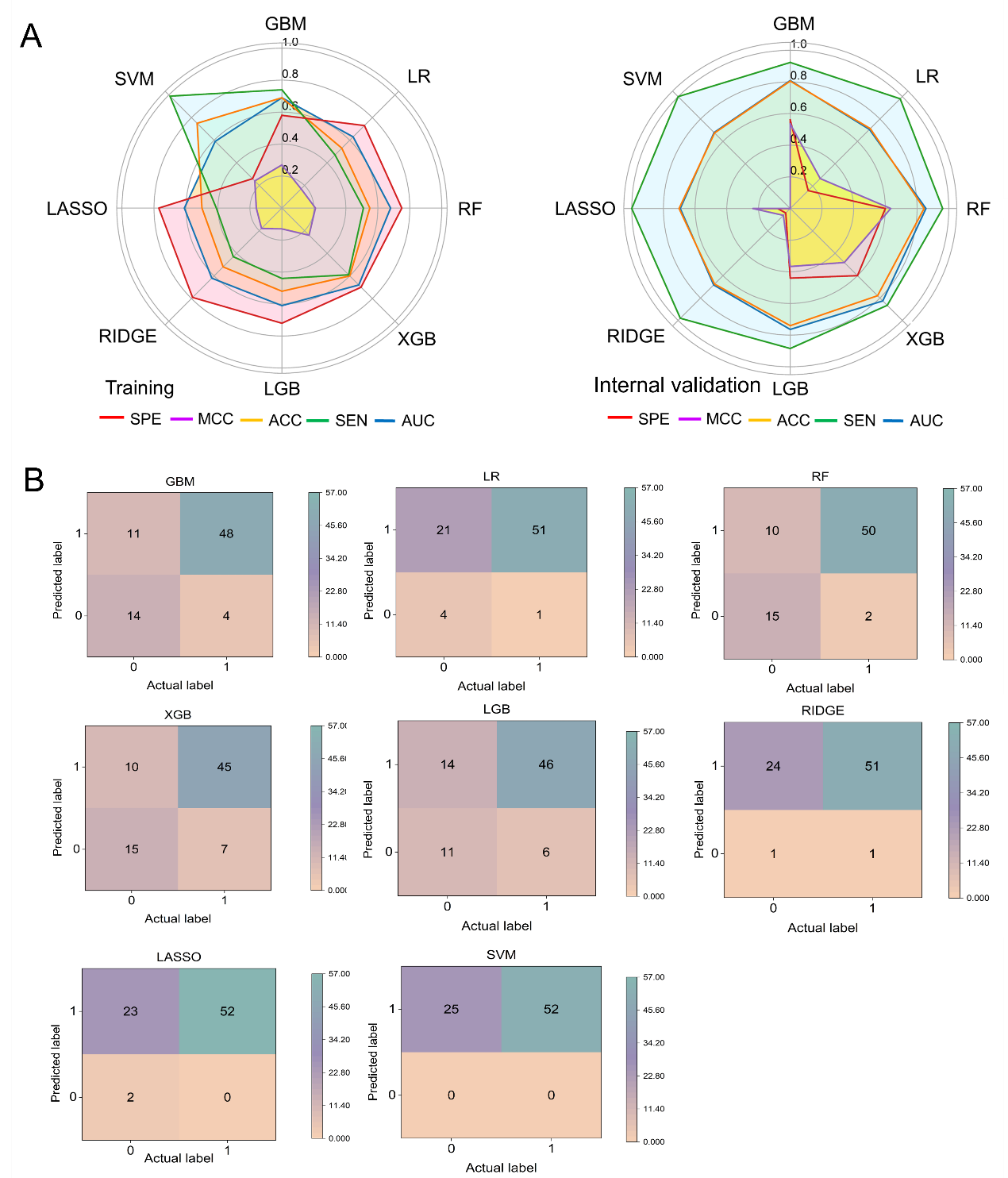
**

**Multimedia Appendix 1.** The performance evaluation of the cold and hot syndrome identification model based on TCM features. A: The performance evaluation’s radar chart of the eight models based on TCM features; B: The confusion matrix analysis of the eight models based on TCM features.
