# Supplementary material for: A Machine Learning Approach to Differentiate Cold and Hot Syndrome in Viral Pneumonia Integrating Traditional Chinese Medicine and Modern Medicine: Machine Learning Model Development and Validation
Source: JMIR Med Inform. 2025 Jul 16;13:e64725. doi: 10.2196/64725 (PMC12286567; doi:10.2196/64725)
Supplement: Multimedia Appendix 5 [file medinform-v13-e64725-s005.docx]

**
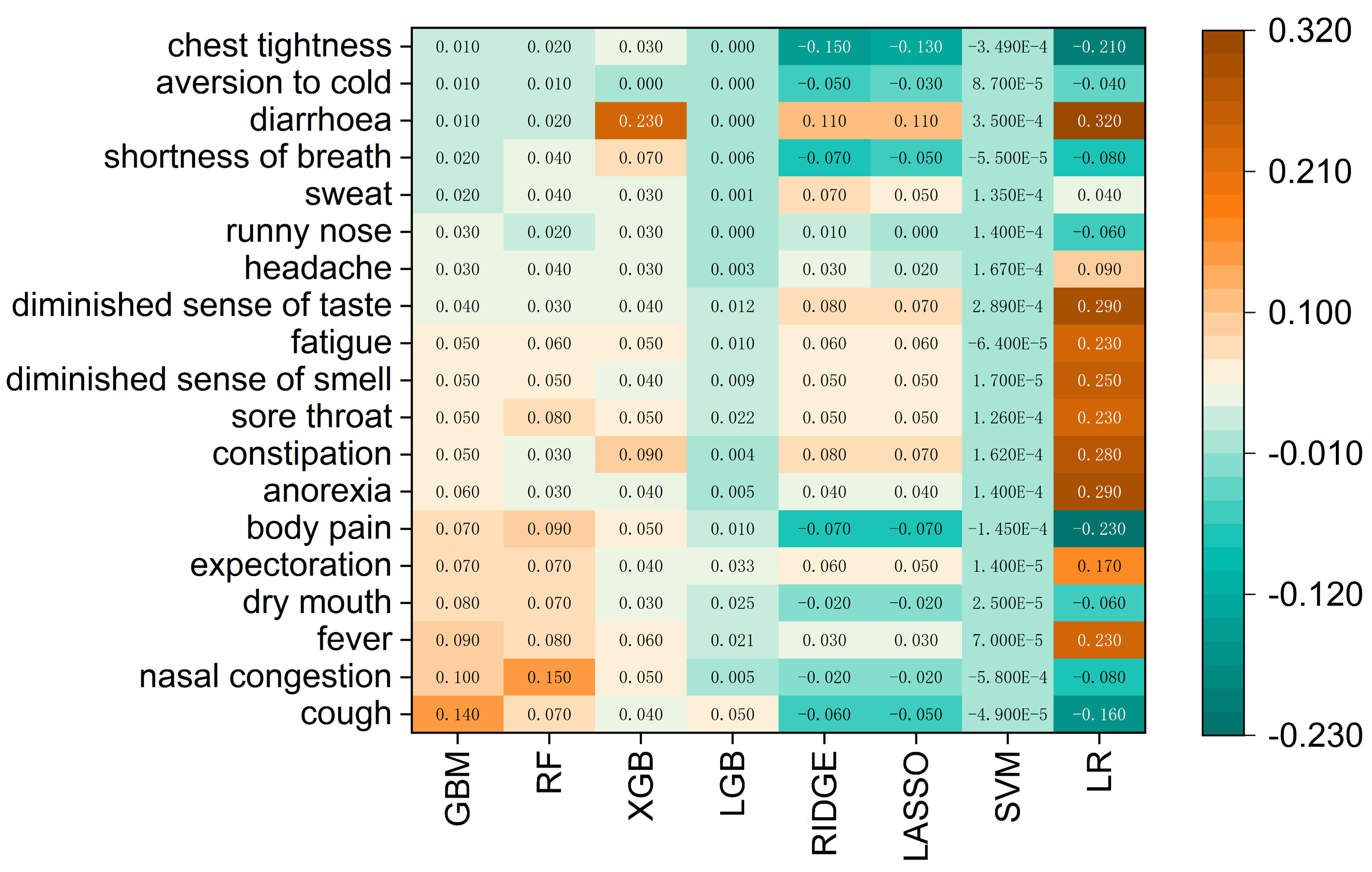
**

**Multimedia Appendix 2.** Heat diagram of TCM features on eight screened models (Note: the value of GBM, RF, XGB, and LGB obtained by feature_importances_. the value of RIDGE, LASSO, SVM, and LR obtained by coef).
